# Experience and lessons from health impact assessment guiding prevention and control of HIV/AIDS in a copper mine project, northwestern Zambia

**DOI:** 10.1186/s40249-017-0320-4

**Published:** 2017-07-04

**Authors:** Astrid M. Knoblauch, Mark J. Divall, Milka Owuor, Kennedy Nduna, Harrison Ng’uni, Gertrude Musunka, Anna Pascall, Jürg Utzinger, Mirko S. Winkler

**Affiliations:** 1 Swiss Tropical and Public Health Institute, P.O. Box, CH-4002 Basel, Switzerland; 2 University of Basel, P.O. Box, CH-4003 Basel, Switzerland; 3 SHAPE Consulting Ltd., GY1 2 St Peter Port, Channel Islands, Guernsey, UK; 4 Solwezi District Health Management Team, 40100 Solwezi, Zambia; 5 First Quantum Minerals Limited, 10100 Lusaka, Zambia

**Keywords:** Community health management, Health impact assessment, HIV, Mining, Occupational health, Sexually transmitted infections, Zambia

## Abstract

**Background:**

To avoid or mitigate potential project-related adverse health effects, the Trident copper project in Kalumbila, northwestern Zambia, commissioned a health impact assessment. HIV was identified a priority health issue based on the local vulnerability to HIV transmission and experience from other mining projects in Africa. Hence, an HIV/AIDS management plan was developed, including community and workplace interventions, with HIV testing and counselling (HTC) being one of the key components. We present trends in HTC data over a 4-year period.

**Methods:**

In 13 communities affected by the Trident project, HTC was implemented from 2012 onwards, using rapid diagnostic tests, accompanied by pre- and post-test counselling through trained personnel. In addition, HTC was initiated in the project workforce in 2013, coinciding with the launch of the mine development. HTC uptake and HIV positivity rates were assessed in the study population and linked to demographic factors using regression analysis.

**Results:**

In total, 11,638 community members and 5564 workers have taken up HTC with an increase over time. The HIV positivity rate in the community was 3.0% in 2012 and 3.4% in 2015, while positivity rate in the workforce was 5.2% in 2013 and 4.3% in 2015. Females showed a significantly higher odds of having a positive test result than males (odds ratio (*OR*) = 1.96, 95% confidence interval (*CI*): 1.55–2.50 among women in the community and *OR* = 2.90, 95% *CI*: 1.74–4.84 among women in the workforce). HTC users in the 35–49 years age group were most affected by HIV, with an average positivity rate of 6.6% in the community sample and 7.9% in the workforce sample. These study groups had 4.50 and 4.95 higher odds of being positive, respectively, compared to their younger counterparts (15–24 years).

**Conclusions:**

While HTC uptake increased five-fold in the community and almost three-fold in the workplace, the HIV positivity rates were insignificantly higher in 2015 compared to 2012. Our data can be used alongside other surveillance data to track HIV transmission in this specific context. Guided by the health impact assessment, the HIV prevention and control programme was readily adapted to the current setting through the identification of socioeconomic and environmental determinants of health.

**Electronic supplementary material:**

The online version of this article (doi:10.1186/s40249-017-0320-4) contains supplementary material, which is available to authorized users.

## Multilingual abstracts

Please see Additional file 1 for translation of the abstract into the six official working languages of the United Nations.

## Background

Extractive sector projects are central for development and economic growth in many low- and middle-income countries [1]. While these projects usually consider potential environmental and social impacts as part of the permitting process, a specific focus on health is often lacking [2–5]. Yet, in tropical countries with high burden of disease, potential health impacts of development projects are a concern [1, 6]. Indeed, there is growing evidence that project developments often cause a broad range of adverse health effects, such as high incidence rates of sexually transmitted infections, pollution of drinking water or elevated transmission of vector-borne diseases [7–9]. In response to these concerns, the health impact assessment (HIA) is the recommended decision-support tool to anticipate and proactively manage project-related health impacts [10–12]. However, there is absence of policy or regulatory capacity in many low- and middle-income countries that require HIA for private sector projects [5].

This holds true for Zambia, which does not have a legislative requirement to assess potential health impacts of infrastructure projects, despite its longstanding history of small- and large-scale mining and associated adverse effects on human wellbeing [5, 13]. For example, the Copperbelt province – the centre of Zambia’s copper mining industry – recorded the highest provincial HIV prevalence in the 2014 Demographic and Health Survey (DHS; 18.2%) compared to 13.3% nationally [14]. Social and economic contexts associated with mining, such as in-migration of mostly young adult males or transactional sex, are believed to have contributed to the high rates of HIV in mining areas, including adjacent villages and along transport corridors [14–17]. To address HIV in this context, the United Nations Development Programme presented an approach of integrating HIV and gender-related issues into the impact assessment process [18].

**Fig. 1:**
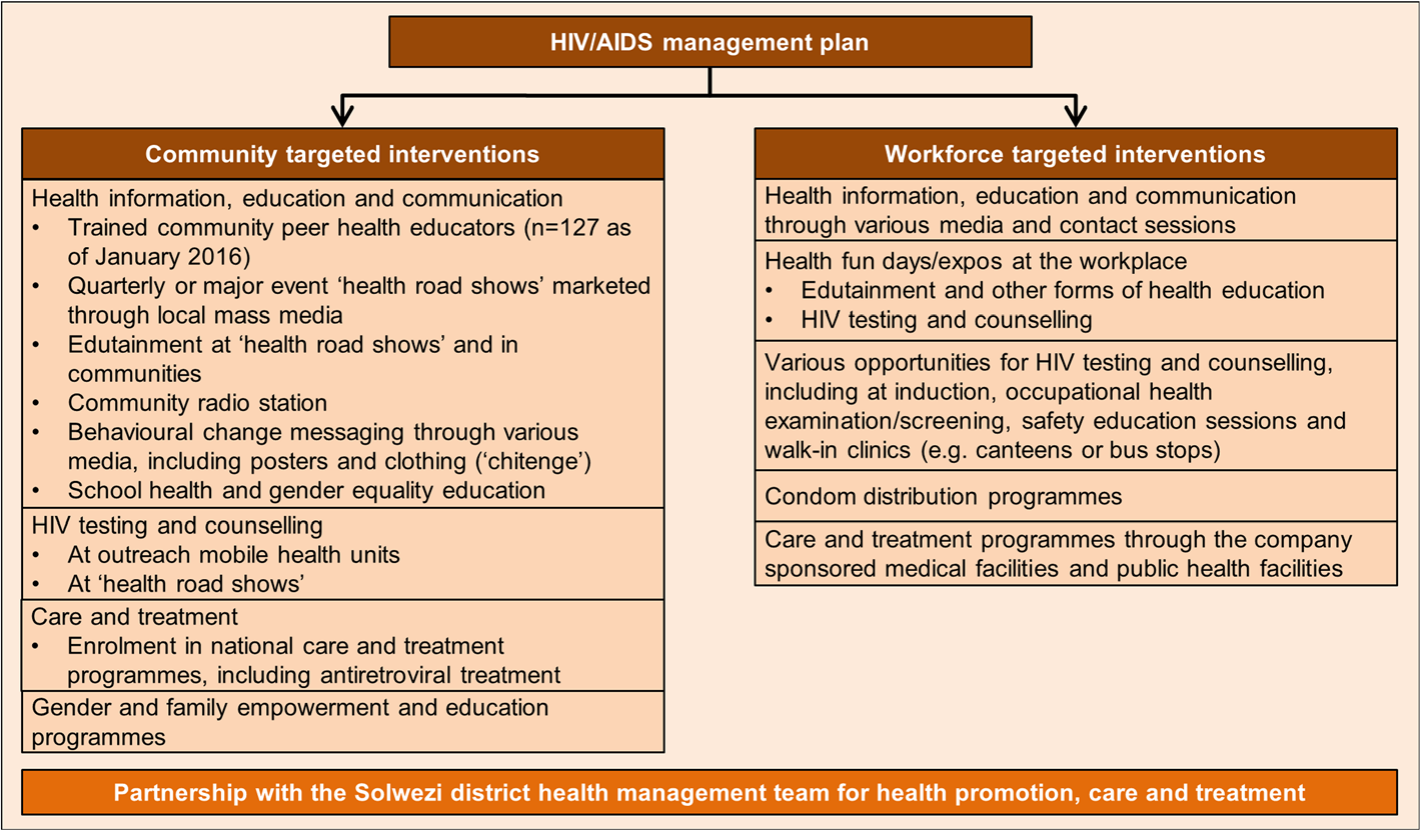
HIV/AIDS management intervention package of the Trident project

The Trident greenfield copper project, located in the Northwestern province of Zambia, operated by First Quantum Minerals Limited (FQML), commissioned an HIA as part of the feasibility studies (2008–2012) [19]. In the HIA scoping phase, secondary data (routine health information system data), qualitative data (obtained from community focus group discussions and key informant interviews with health staff) and quantitative data from a cross-sectional baseline health survey were employed [20–22]. Potential health impacts were predicted using a standardised, semi-quantitative risk assessment tool [23]. The transmission of sexually transmitted infections, including HIV/AIDS, was identified as a priority, given the existing community health risks in project-affected communities and precedence in other mining areas [24]. Qualitative data obtained in the frame of the HIA scoping study showed that the local communities are vulnerable to sexually transmitted infections for a number of reasons. First, the low estimated baseline prevalence rate of sexually transmitted infections might rapidly change in face of in-migration from high-prevalence areas. Second, the relatively high socioeconomic vulnerability could encourage women to engage in transactional sex. Third, at the onset of the project, there was little exposure to preventive interventions. Fourth, the available data suggested inequities in access to health care. Fifth, the use of condoms was low, coupled with women’s weak negotiating power. Finally, we noted high stigma associated with sexually transmitted diseases, particularly HIV [21]. Hence, as a result of the HIA, the Trident project, in partnership with the Solwezi district health management team, initiated an HIV intervention package. Figure 1 outlines the interventions in both the community and the workforce. Health promotion activities, such as peer education, radio programmes, information, education and communication (IEC) and empowerment campaigns in communities and schools, were implemented. HIV testing and counselling (HTC) was the key feature of the programme, which was offered in communities through specific outreach activities and in the workplace on a continuous basis at various service points. Care and treatment enrolment rounded up the intervention package. The interventions were adapted to the local context and were implemented right from the onset of the mine development.

Here, we present the approaches and outcomes of the community- and workforce-based HTC implemented in this particular copper mining setting over a 4-year period. The objectives of reporting these data are (i) to present HTC uptake and HIV positivity rate over a 4-year period in the workforce and communities affected by a large mining project in sub-Saharan Africa; (ii) to provide supplementary data for national HIV surveillance; and (iii) to provide a publicly available good practice example of HIA and associated health risk mitigation and monitoring. Experience and lessons of HIA in anticipating and managing risks associated with HIV are discussed.

## Methods

### Study area

**Fig. 2:**
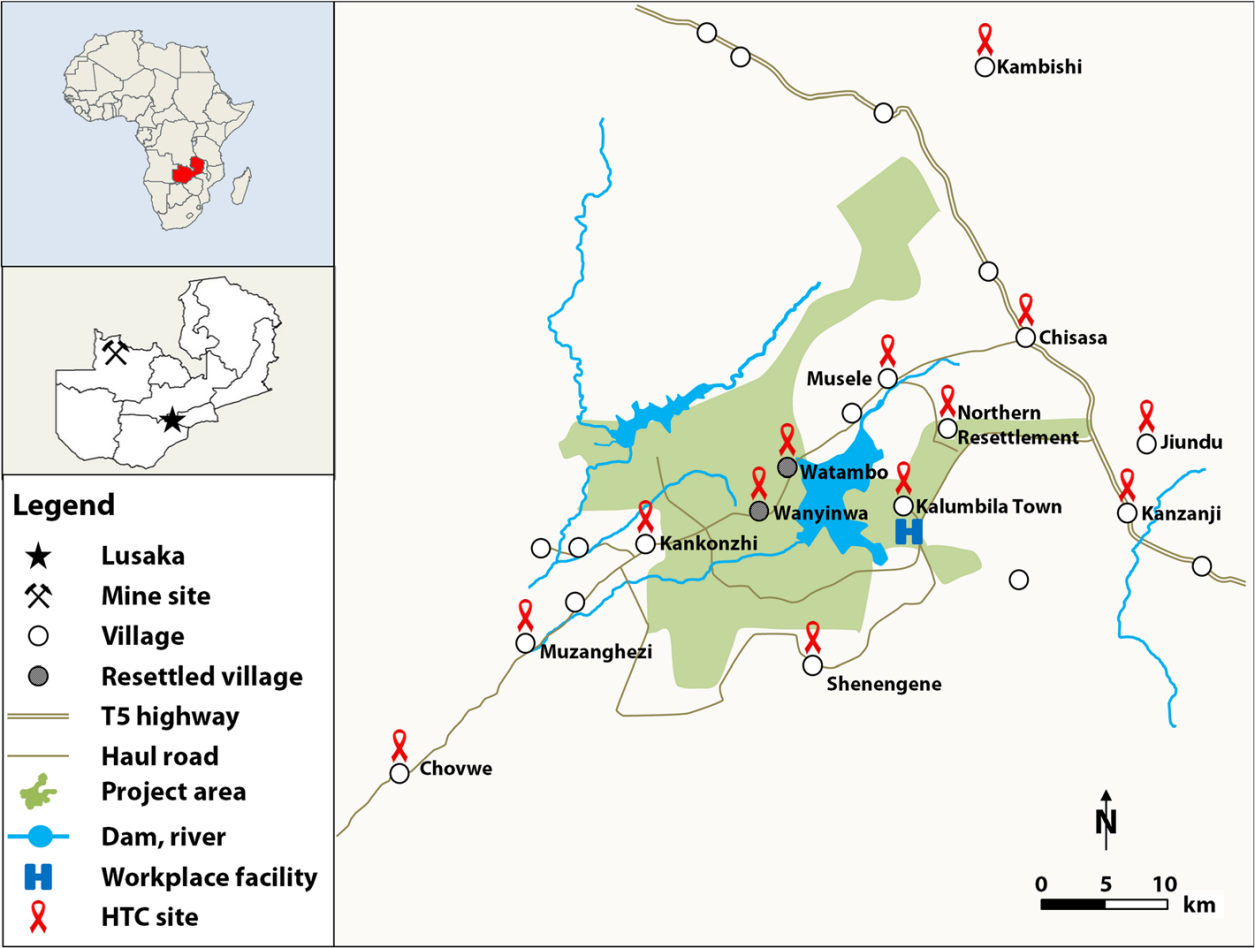
Trident project location and perimeter of the HIV intervention package

The Trident project is located in a rural area in Solwezi district, approximately 150 km northwest of Solwezi, the provincial capital (Fig. 2) [19]. The mine development transformed the local environment (e.g. open pit mine, damming of rivers and construction of new roads), caused population movements (e.g. resettlement and in-migration) and a shift of local occupational activities [25–27].

### Study design and study population

The HTC was offered in communities as mobile outreach activities. Hence, each community visit represents a cross-sectional sample. For analysis, results were pooled over 1-year periods and stratified by community. In 2012, the HTC programme was launched in six communities and gradually expanded to cover as many as 13 communities by 2015 (Fig. 2). For the workforce, HTC was offered continuously at various service points that were readily accessible by mine workers (e.g. work-based health facility, bus stops and canteens). Both community members and workers were offered HTC through a voluntary walk-in concept without any selection or randomisation process being applied.

### HIV testing

HIV testing was performed following standardised procedures defined by the Zambian Ministry of Health, using two types of approved HIV rapid diagnostic test (RDT) kits: Alere Determine™ HIV-1/2 (Alere Medical Co. Ltd.; Matsudo, Japan) and UniGold HIV (Trinity Biotech Manufacturing Ltd.; Bray, Ireland), detecting HIV 1/2 antibodies in capillary blood samples. A negative RDT test was considered HIV-negative, while a positive result required a second confirmatory test. A second positive test was considered HIV-positive, with a negative second test reported as an intermediate result, requiring further testing. Results were promptly communicated to the participants as part of post-test counselling [28].

### Data recording and statistical analysis

A standardised form was completed for each participant, including basic demographic information, residential location, occupation, reason prompting the test, any previous tests and test result. The form contained no identifying features other than a unique number linked to a register that was stored securely. Data were entered into EpiData version 1.4.4 (EpiData Association; Odense, Denmark) and analysed in Stata version 13 (StataCorp LP; College Station, USA). Descriptive analysis and multivariate logistic regression were performed to obtain HIV positivity rates and odds ratio (*OR*) statistics, including 95% confidence intervals (*CI*s). Gender, age, marital status, year and type of tester (first-time or repeat) were adjusted for in the regression model as they are either known determinants of HIV infection or included in order to identify higher risk groups [14].

### Ethical considerations

Pre-test counselling and written informed consent were obtained from all participants prior to the collection of a capillary blood sample to test for HIV. Post-test counselling was performed for all positive and negative results, with participants who tested HIV positive managed in accordance with the Zambian National AIDS Strategic Framework 2011–2015, including referral for registration, determination of CD4 count and enrolment in care and treatment programmes [28]. Cases enrolled for care and treatment in the workforce or their dependents were followed up at the workplace health facility [28].

## Results

### HTC uptake

HTC uptake is derived from the study population size. In the community, a total of 11,638 individuals, aged 15–49 years, have tested for HIV between 2012 and 2015. Invalid records (e.g. being out of the age range analysed here) were excluded. Background characteristics of the study population per year are shown in Table 1. The majority of testers in the communities were female (>58% in all four years), married or in a long-term relationship (>57% in all four years) and about half (range 48.7–56.5% ﻿﻿﻿over the four years﻿﻿) were from the youngest age group (15–24 years).

**Table 1:** Characteristics of community and workplace HTC users aged 15–49 years, Trident project area, 2012–2015

Year	2012	2013	2014	2015
Community (*n*)	1003	2818	1853	5964
Males (*n*; %)	365 (36.4)	1028 (36.5)	774 (41.8)	2261 (37.9)
Females (*n*; %)	638 (63.6)	1790 (63.5)	1079 (58.2)	3703 (62.1)
Average age in years ± SD	25.1 ± 7.9	26.3 ± 8.4	26.3 ± 7.8	26.2 ± 8.3
15–24 years old (*n*; %)	567 (56.5)	1457 (51.7)	902 (48.7)	3089 (51.8)
25–34 years old (*n*; %)	295 (29.4)	835 (29.6)	636 (34.3)	1775 (29.8)
35–49 years old (*n*; %)	141 (14.1)	526 (18.7)	315 (17.0)	1100 (18.4)
Married or in long-term relationship (%; 95% *CI*)	57.9 (54.8–61.0)	61.3 (59.4–63.1)	63.6 (61.3–65.8)	66.6 (65.4–67.8)
First-time tester (%; 95% *CI*)	33.5 (30.5–36.5)	32.7 (31.0–34.5)	24.8 (22.8–26.9)	25.5 (24.4–26.7)
Repeat tester (%; 95% *CI*)	66.5 (63.5–69.5)	67.3 (65.5–69.0)	75.2 (73.1–77.2)	74.5 (73.3–75.6)
Average overall HIV positivity rate (%; 95% *CI*)^1^	3.0 (2.0–4.2)	3.3 (2.6–3.9)	3.4 (2.5–4.2)	3.4 (2.9–3.9)
Workforce (*n*)	-	1011	1852	2701
Males (*n;* %)	-	980 (96.9)	1753 (94.7)	2555 (94.6)
Females (*n*; %)	-	31 (3.1)	99 (5.4)	146 (5.4)
Average age in years ± SD	-	30.9 ± 7.3	30.7 ± 6.7	31.7 ± 6.8
15–24 years old (*n*; %)	-	213 (21.1)	352 (19.0)	396 (14.7)
25–34 years old (*n*; %)	-	487 (48.2)	995 (53.7)	1413 (52.3)
35–49 years old (*n*; %)	-	311 (30.8)	505 (27.3)	892 (33.0)
Married or in long-term relationship (%; 95% *CI*)	-	77.9 (75.3–80.5)	77.4 (75.4–79.3)	89.0 (87.7–90.1)
First-time tester (%; 95% *CI*)	-	39.2 (36.2–42.3)	41.4 (39.1–43.8)	11.8 (10.5–13.1)
Repeat tester (%; 95% *CI*)	-	60.8 (57.7–63.8)	58.6 (56.2–60.9)	88.2 (86.9–89.5)
Average overall HIV positivity rate (%; 95% *CI*)^1^	-	5.2 (4.0–6.8)	3.5 (2.7–4.5)	4.4 (3.6–5.2)

*CI*, confidence interval; *SD* standard deviation; −, no data available

^1^Samples of individual years are non-independent due to repeat testers (although not all repeat testers necessarily tested more than once in the HTC offered by FQML) leading to a too narrow *CI* estimate

For the workforce, data were available from 2013 to 2015 with an overall study population of 5564 workers (aged 15–49 years). More than 90% of the participants were males, reflecting the typical gender ratio in mining sites (Table 1). The average age was around 31 years with about half of the participants (range 48.2–53.7%) in the 25–34 year-old age group.

**Fig. 3:**
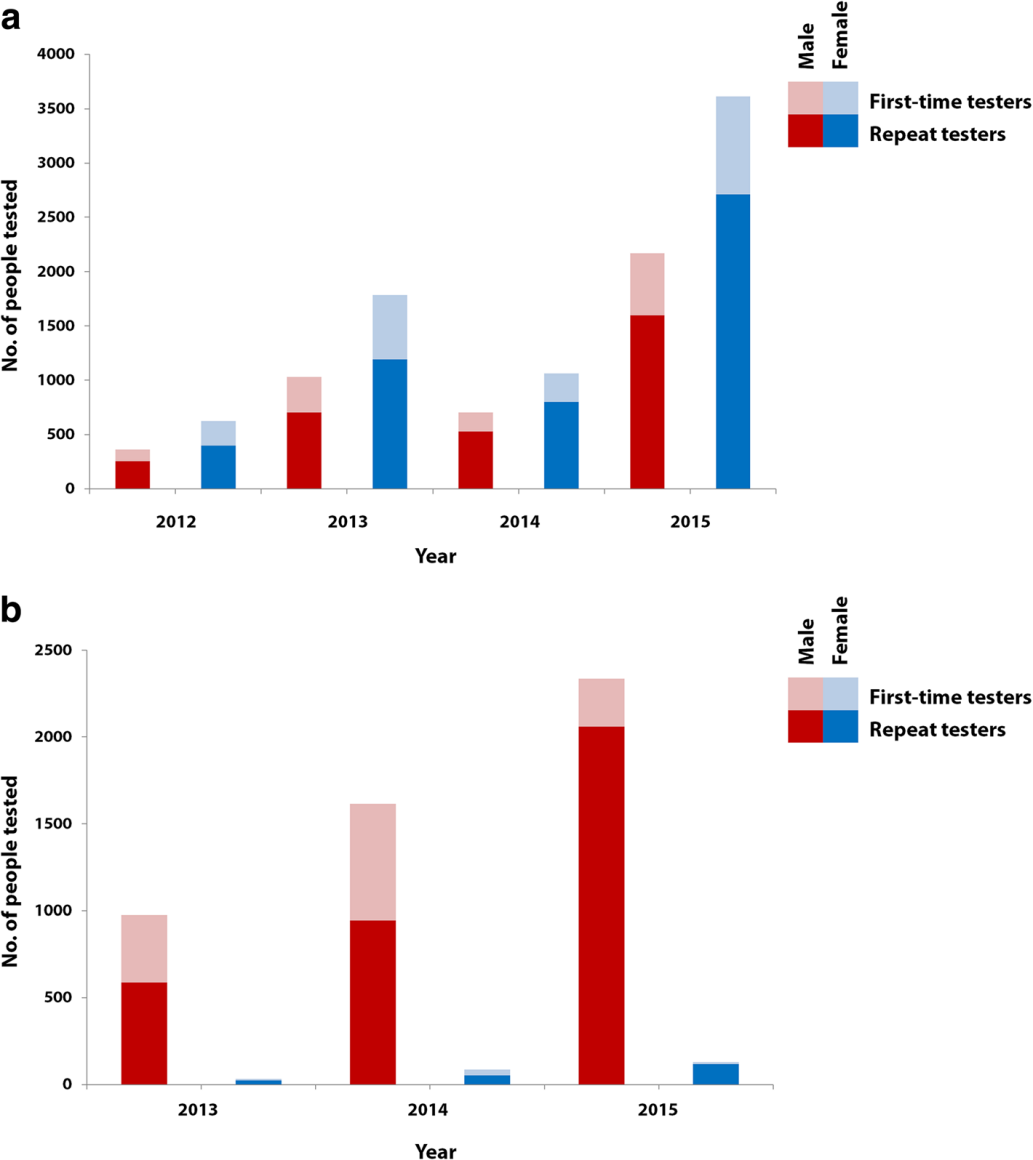
Uptake of HTC by first-time and repeat testers aged 15–49 years, Trident project area, 2012-2015. **a** Community; **b** workforce

The uptake of HTC in the general community and the workforce, stratified per year, is illustrated in Fig. 3a and 3b, including a differentiation between first-time and repeat testers. The number of community participants increased from 2012 to 2013, decreased in 2014 and was highest, both in males and females, in 2015. Two-thirds of HTC users were repeat testers in 2012 and 2013. This proportion increased to 75% in 2014 and 2015 (Table 1).

In the workforce, uptake continuously increased in males and females. The proportion of first-time testers was comparable in 2013 and 2014 at around 40%. It decreased markedly to 11.8% in 2015.

In both the community and the workforce sample, the main reasons to test were ‘just to know’ and ‘just to make sure’, as stated by >90% of males and females (Table 2). Few individuals stated other reasons, such as ‘feeling sick’ or ‘worried about partner behaviour.

**Table 2:** Self-reported reasons for using HTC services in adults aged 15–49 years, Trident project area, 2012–2015

Reasons stated for HTC use (%)	Community		Workforce	
Gender	Male	Female	Male	Female
*n*	4224	6867	5035	261
Feeling sick	0.4	0.4	0.1	0.0
Partner is HIV-positive	0.1	0.1	0.1	0.0
Expecting parent, potential prevention of mother-to-child transmission	0.3	3.5	0.0	0.0
Just to make sure	22.0	22.4	31.6	31.0
Just to know	77.1	73.2	68.0	68.2
Worried about partner behaviour	0.1	0.2	0.1	0.4
Other	0.1	0.1	0.1	0.4

### HIV positivity rate

**Fig. 4:**
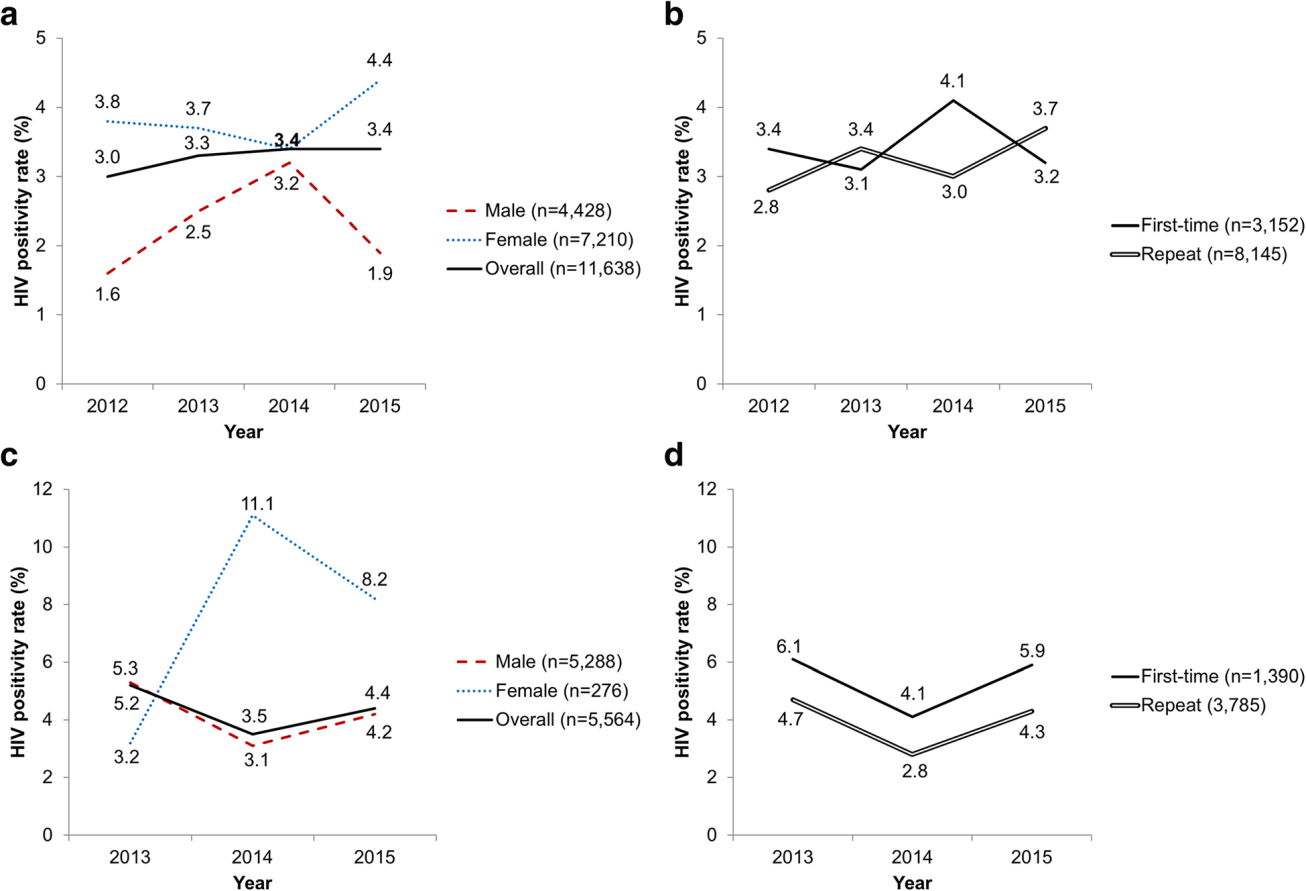
HIV positivity rate in HTC users aged 15–49 years, Trident project area, 2012–2015. **a** Community, stratified by sex; **b** community, stratified by first-time and repeat testers; **c** workforce, stratified by sex; **d** workforce, stratified by first-time and repeat testers

In the community sample, the overall HIV positivity rate was slightly higher in 2014 and 2015 (3.4% in both years) than the previous years (3.0% in 2012 and 3.3% in 2013; Fig. 4). As shown by logistic regression in Table 3, there was no significant change over time. It needs to be noted that samples of individual years are not independent due to potential repeat testers (although not all repeat testers necessarily tested more than once in the HTC offered by FQML), leading to too narrow *CI* estimates. The HIV positivity rate in females was consistently higher than in males. Overall, females had twice the odds of being tested positive for HIV than males (*OR* = 1.96, 95% *CI*: 1.55–2.50). No significant difference was found in the positivity rate between first-time (3.3%) and repeat testers (3.3%). The ORs of testing positive were significantly higher in the older age groups (i.e. 25–34 and 35–49 years), compared to the youngest age group (15–24 years). Their ORs to test positive were 2.61 (95% *CI*: 2.00–3.40) and 4.50 (95% *CI*: 3.43–5.90), respectively (Table 3).

**Table 3:** Multivariate logistic regression analysis for the HIV positivity rate in HTC users aged 15–49 years, Trident project area, 2012–2015

		Community			Workforce		
		*n*	Positivity rate 2012–2015 (%, 95% *CI*)^a^	a*OR* ^b^ (95% *CI*)	*n*	Positivity rate 2013–2015 (%, 95% *CI*)^a^	a*OR* ^b^ (95% *CI*)
Gender	Male	4428	2.3 (1.8–2.7)	1.00	5288	4.0 (3.4–4.5)	1.00
	Female	7210	4.0 (3.5–4.5)	1.96 (1.55–2.50)	276	8.7 (5.6–12.7)	2.90 (1.74–4.84)
Year	2012	1003	3.0 (2.0–4.2)	1.00	n/a	n/a	n/a
	2013	2818	3.3 (2.6–3.9)	1.03 (0.67–1.58)	1011	5.2 (3.9–6.8)	1.00
	2014	1853	3.4 (2.5–4.2)	1.10 (0.69–1.73)	1852	3.5 (2.7–4.4)	0.61 (0.41–0.90)
	2015	5964	3.4 (2.9–3.9)	1.11 (0.75–1.67)	2701	4.3 (3.6–5.2)	0.89 (0.62–1.27)
Type	Repeat tester	8145	3.3 (2.9–3.7)	1.00	3785	4.0 (3.3–4.6)	1.00
	First-time tester	3152	3.3 (2.7–3.9)	1.03 (0.82–1.31)	1390	5.0 (3.9–6.3)	1.48 (1.08–2.03)
Marital status	Married or long-term relationship	7457	3.4 (2.9–3.8)	1.00	4624	4.3 (3.7–4.9)	1.00
	Other	4181	3.3 (2.7–3.8)	1.46 (1.17–1.84)	940	3.9 (2.7–5.3)	1.22 (0.80–1.84)
Age group	15–24 years	6015	1.8 (1.4–2.1)	1.00	961	2.0 (1.1–3.0)	1.00
	25–34 years	3541	4.0 (3.4–4.7)	2.61 (2.00–3.40)	2895	2.8 (2.2–3.5)	1.63 (0.96–2.75)
	35–49 years	2082	6.6 (5.5–7.7)	4.50 (3.43–5.90)	1708	7.9 (6.6–9.2)	4.95 (2.94–8.32)

a*OR*, adjusted odds ratio; *CI*, confidence interval; *n/a*, not applicable

^a^Samples of individual years are non-independent due to repeat testers (although not all repeat testers necessarily tested more than once in the HTC offered by FQML) leading to a too narrow CI estimate

^b^ *OR* are mutually adjusted for the variables listed in the table

Trends in the HIV positivity rate, stratified by communities over the 4-year period, are shown in Table 4. In 2015, HIV positivity rates were highest in the communities of Northern Resettlement (5.1%), followed by Chisasa (5.0%) and Kanzanji (4.8%).

**Table 4:** HIV positivity rate in community members aged 15–49 years using HTC services per community, Trident project area, 2012–2015

Community	2012		2013		2014		2015	
	*n*	% (95% *CI*)^a^	*n*	% (95% *CI*)^a^	*n*	% (95% *CI*)^a^	*n*	% (95% *CI*)^a^
Kankonzhi	117	4.3 (1.4–9.6)	390	1.8 (0.7–3.6)	281	2.1 (0.7–4.5)	600	3.0 (1.7–4.6)
Wanyinwa	240	4.6 (2.3–8.0)	455	4.0 (2.3–6.1)	-	-	-	-
Musele	270	1.9 (0.6–4.2)	753	3.9 (2.5–5.4)	347	1.7 (0.6–3.7)	1123	3.0 (1.7–4.6)
Chisasa	245	3.3 (1.4–6.3)	621	3.4 (2.1–5.1)	474	3.6 (2.1–5.6)	1193	5.0 (3.8–6.4)
Chovwe	74	1.4 (0.0–7.3)	311	2.3 (0.9–4.5)	156	4.5 (1.8–9.0)	563	4.3 (2.7–6.2)
Muzangezhi	57	0.0 (.-.)	-	-	-	-	226	1.7 (0.4–4.4)
Watambo	-	-	288	3.5 (1.6–6.2)	-	-	220	0.5 (0.0–2.5)
Northern Resettlement	-	-	-	-	255	3.5 (1.6–6.5)	415	5.1 (3.1–7.6)
Kalumbila Town	-	-	-	-	340	5.0 (2.9–7.8)	471	3.8 (2.2–5.9)
Shenengene	-	-	-	-	-	-	425	2.1 (0.9–3.9)
Kanzanji	-	-	-	-	-	-	230	4.8 (2.4–8.3)
Jiundu	-	-	-	-	-	-	336	0.6 (0.0–2.1)
Kambishi	-	-	-	-	-	-	162	1.2 (0.1–4.3)
Overall	1003	3.0 (2.0–4.2)	2818	3.3 (2.6–3.9)	1853	3.4 (2.5–4.2)	5964	3.4 (2.9–3.9)

CI, confidence interval; -, no data available

^a^Samples of individual years are non-independent due to repeat testers (although not all repeat testers necessarily tested more than once in the HTC offered by FQML) leading to a too narrow *CI* estimate

In the workforce, the HIV positivity rate in males was 5.3% in 2013, 3.1% in 2014 and 4.2% in 2015 (Fig. 4c). An upward trend was observed in the positivity rate in females: one out of 31 (3.2%) women tested positive in 2013; 11 out of 99 (11.1%) in 2014 and 12 out of 146 (8.2%) in 2015. Similar to the community sample, females had almost three times higher odds of a positive HIV test (*OR* = 2.90, 95% *CI*: 1.74–4.84; Table 3). Across all years, first-time testers in the workforce sample had a significantly higher HIV positivity rate than repeat testers (5.0% *vs*. 4.0%, *OR* = 1.48, 95% *CI*: 1.08–2.03). The HIV positivity rate was strongly dependent on the age of workers, as those aged 35–49 years had an almost 5 times higher odds of a positive test compared to the youngest age group (*OR* = 4.95, 95% *CI*: 2.94–8.32).

## Discussion

The findings from HTC from 2012 to 2015 offered in the Trident project mining area suggest a growing trend of HTC uptake, an insignificantly higher HIV positivity rate in 2014/2015 compared to earlier years (2012 and 2013) in the general community and a insignificantly lower rate in the workplace population in 2015 (4.4%) compared to 2013 (5.2%).

### HTC uptake

HTC is one of the key strategies for HIV control but to date, most people in low- and middle-income countries still do not know their HIV status [29, 30]. In Zambia, HTC uptake is promoted at different venues, including facility-, community- and workplace-based HTC [28, 31]. As was previously found in rural communities with weak health infrastructure and HIV programming, the provision of community-based HTC in the 13 communities supported the uptake of HIV testing [32–34]. Since the HTC described here is provider-driven, this can be partly attributed to the extension of activities into newly visited communities and the up-scaling of activities in previously visited communities and partly to an increased demand. In two repeated cross-sectional surveys in seven project-impacted communities, it was found that the proportion of females who have ever performed an HIV test increased from 76.6% in 2011 to 86.1% in 2015 [20, 27]. At the workplace, uptake augmented uniformly every year. Convenient access to HTC at multiple workplace locations was previously found to increase HIV testing coverage, especially when linked to HIV care and treatment programmes [35, 36]. In 2015, Solwezi district had by far the highest uptake of HTC, accounting for 55% of the total of 56,902 HTC users in Northwestern province [37]. In both the workplace and community study populations, the proportion of repeat testers increased over time, as in other HTC studies, suggesting an effective programme [34, 38].

### HIV positivity rate

The HIV positivity rate measured through community-based HTC was slightly higher in 2015 (3.4%) than in 2012 (3.0%). This is lower than prevalences measured during the 2013-2014 DHS in Northwestern province in the same age group (6.4%), although comparability with the non-random estimates presented here is limited [14, 39, 40]. Hence, these observations must be interpreted against the following background. First, there is a presumably lower HIV prevalence in the rural study area pre-project, as compared to other areas in the province (e.g. urban centres or mining towns) [14]. Second, the highest HIV prevalence during the DHS was observed in mining towns [17, 41]. Third, we noted targeted HIV management in the study area, in face of potential exclusion of high prevalence groups. Importantly, HTC in our setting was provider- rather than demand-driven, which might explain some of the observed differences over time and space. The highest HIV positivity rates in 2015 were recorded in Northern Resettlement and Chisasa (5.1% and 5.0%, respectively). These communities are dynamic, with extensive in- and out-migration. Northern Resettlement has attracted job-seeking migrants with a high proportion currently employed (in July 2015, 76.5% of households had at least one member employed by the project). This has led to relative wealth in the community compared to the surrounding rural areas. Chisasa is the main urban settlement outside of the project area and has received the bulk of migrants, with a cross-sectional health survey conducted in mid-2015 reporting that 68% of respondents were resident for less than 5 years [15]. Moreover, Chisasa reported the highest rates of multiple sex partners and transactional sex in the 12 months before the said survey [17]. Data from this study, together with other social and health data, support the identification of hotspot communities and the timely design of preventive measures tailored in response to the needs of these communities.

The overall HIV positivity rate in the workforce was consistently 1–2% above the rate observed in the communities. The effect of labour migration from areas of high HIV prevalence on the observed HIV positivity rates is difficult to assess as participants’ migration history was not inquired prior to 2016 [16, 40]. In 2015, 9.7% of people aged 15 years and above were tested positive for HIV during HTC in Solwezi district [37]. These data mainly stem from mining towns, such as Solwezi. Hence, the higher HIV positivity rate in the workforce might be explained by the higher HIV prevalence observed in these labour-sending areas [16, 37]. Whilst the observed trends in HIV positivity rates have not shown significant increases as seen in other mining areas in Africa, rigorous surveillance is warranted to rapidly pick up changing patterns and high-risk groups [42, 43].

Our reported HIV positivity rate data do not shed full light on the true prevalence or incidence. Nevertheless, our data may serve as a benchmark for surveillance in this population affected by a large mining project complementary to other data sources, such as the Zambia national sentinel surveillance system and population-based prevalence surveys [14, 44].

### Experience from HIA

Ending the AIDS epidemic by 2030 is a declared goal in the Sustainable Development Goals (SDGs) era [45]. According to the SDGs agenda, health-related targets are to be considered in developments across all sectors [46]. The HIA provides a means to monitor health and determinants of health indicators and therefore accountability towards the general public, whilst at the same time creating inter-sectoral collaboration and strengthening public-private partnerships. For the Trident project, the HIA guided, through the identification of socioeconomic and environmental determinants of health, the HIV management plan, which was readily adapted to the specific context and supported the Zambian HTC implementation plan, while at the same time mitigating project-related risks [31]. According to experience by project decision-makers, the implementation of the HIV management plan was further promoted through: (i) the continuous support of the HIA by public health professionals; (ii) the close collaboration with the Ministry of Health at national and local level; (iii) adequate and skilled human resources to implement the interventions; (iv) the resident model, i.e. national migrant and expatriate workers reside with their families in the area, as opposed to the rotational model with frequent travel into and out of the area; (v) the consistency of funding and management support from FQML; and, importantly (vi) the partnership and support from traditional authorities and local communities.

### Limitations

First, the data presented here stem from HTC offered on a voluntary basis (i.e. walk-in mobile clinics). Hence, our approach was provider- rather than demand-driven, which might lead to under- or over-representation of particular population groups. Second, community members and workers may use alternative facilities for HTC or test more than once, and hence, the reported HTC uptake and HIV positivity rate might be slightly under- or overestimated. Third, our data do not allow distinguishing between first-time or repeat testers offered by FQML or elsewhere. Due to repeat testers within the FQML HTC, the samples of individual years are non-independent, leading to too narrow CI estimates. Of note, in the Additional file 2, we show HIV positivity rates and CI for first-time testers only. Finally, the HIV positivity rate data in both study populations presented are monitoring trends but caution is warranted when relying on these for estimation of prevalence or detection of sudden changes in transmission, due to the time lag between exposure and testing date and the extent of non-testers.

## Conclusions

Between 2012 and 2015, HTC uptake increased five-fold in the community and almost three-fold in the workforce study populations in the Trident project area in Zambia, whilst the HIV positivity rates was insignificantly higher in 2015 compared to 2012. In the current mining setting, the HIA triggered the continued collection of data which can be used along with other surveillance data sources in tracking HIV trends. There are few examples in the public domain on how HIV/AIDS and other health issues are addressed within an evidence-based impact assessment process for mining or other large-scale projects in sub-Saharan Africa [22, 47, 48]. Good practice case studies on how HIA and associated health monitoring can benefit communities, the host government and projects are needed to increase its visibility and underpin the importance of institutionalizing HIA at the national level in low- and middle-income countries [5, 18]. The current study has several strengths. First, it allows to transparently communicate trends in HTC uptake and HIV positivity rate in the copper mine project area with the public. Second, hotspot-communities that need to be intensively targeted with preventive interventions could be identified. The research effort can serve as a good practice example of HIA and inter-sector collaboration, as advocated by the 2030 SDGs agenda.

## Additional files

Additional file 1: Multilingual abstracts in the six official working languages of the United Nations. (PDF 639 kb)

Additional file 2: HIV positivity rate of first-time testers in community members and workforce aged 15–49 years, Trident project area, 2012–2015. (DOCX 44 kb)

## Abbreviations

AIDS: Acquired Immune Deficiency Syndrome
CI: Confidence Interval
DHS: Demographic and Health Survey
FQML: First Quantum Minerals Limited
HIA: Health Impact Assessment
HIV: Human Immunodeficiency Virus
HTC: HIV Testing and Counselling
OR: Odds Ratio
RDT: Rapid Diagnostic Test
WHO: World Health Organization
