# Supplementary material for: Experience and lessons from health impact assessment guiding prevention and control of HIV/AIDS in a copper mine project, northwestern Zambia
Source: Infect Dis Poverty. 2017 Jul 4;6:114. doi: 10.1186/s40249-017-0320-4 (PMC5496403; doi:10.1186/s40249-017-0320-4)
Supplement: Supplementary file 1 — Multilingual abstracts in the six official working languages of the United Nations. (PDF 639 kb) [file 40249_2017_320_MOESM1_ESM.pdf]

Translation of the abstract into the six official working languages of the United Nations

ألخبرة والدروس المستمدة من تقييم ألتأثير الصحي للوقاية والسيطرة على فيروس نقص المناعة البشرية / الإيدز في مشروع منجم للنحاس في شمال غرب زامبيا

خلاصة البحث

ألخلفية العلمية : لتجنب أو تخفيف الآثار الصحية الضارة المحتملة للمشروع ، أجرى مشروع ثلاثي المحور للنحاس في كولومبيا، شمال غرب زامبيا، لتقييم الأثر الصحي في مشروع النحاس ثلاثي المحور. تم تحديد فيروس نقص المناعة البشرية وهومن القضايا الصحية ذات الأولوية على أساس الضعف المحلي لنقل فيروس نقص المناعة البشرية والخبرة السابقة من مشاريع التعدين الأخرى في أفريقيا. لهذا السبب، وضعت خطة لإدارة فيروس نقص المناعة البشرية / الإيدز، بما في ذلك المجتمع وتداخلات أماكن العمل، حيث يعتبر اختبار فيروس نقص المناعة البشرية وإسداء المشورة له أحد المكونات الرئيسية.وبتنفيذ هذا الأختبار وتقديم المشورة لحالات ألأيدز(HTC) كونها واحدة من المكونات الرئيسية. نقدم الاتجاهات في بيانات أختبار وتقديم المشورة لحالات ألأيدز على مدار 4 سنوات.

طرق البحث: في 13 مجتمعا متأثرا بالمشروع الثلاثي المحور ، نفذ أختبار وتقديم المشورة لحالات ألأيدز منذ عام 2012 فصاعدا، باستخدام اختبارات التشخيص السريع، مصحوبا بالمشورة قبل وبعد الاختبار من خلال الموظفين المدربين. فضلا عن ذلك، بدأ أختبار وتقديم المشورة لحالات ألأيدز في القوى العاملة المشاريع في عام 2013، بالتزامن مع إطلاق تطوير المناجم. تم تقييم أختبار وتقديم المشورة لحالات ألأيدز و معدلات الإيجابية في مجتمع الدراسة وربطها بالعوامل الديموغرافية باستخدام تحليل الانحدار.

ألنتائج: إجمالا، خضع 11،638 من أفراد المجتمع و 5،564 عاملا لأختبار وتقديم المشورة لحالات ألأيدز مع زيادة مع مرور الوقت. وكانت نسبة الحالات الموجبة لفيروس نقص المناعة البشري في المجتمع 3.0 % في عام 2012 و 3.4% في عام 2015، في حين بلغت نسبة الحالات الموجبة للفايروس في القوى العاملة 5.2 % في عام 2013 و 4.3 % في عام 2015. أظهرت النتائج أن نسبة الحالات الموجبة بين النساء كانت عالية (نسبة االأحتمالية = 1.96، 95٪ فاصل الثقة 2.50-1.55 بين النساء في المجتمع و نسبة االأحتمالية = 2.90، 95٪ فاصل الثقة 1.74-4.84 بين النساء في القوى العاملة). تبين أن مستخدمو أختبار وتقديم المشورة لحالات ألأيدز من الفئة العمرية 35-49 سنة الأكثر تأثرا بفيروس نقص المناعة البشرية، بنسبة موجبة 6.6٪ في عينة المجتمع و 7.9٪ في عينة القوى العاملة. وكانت احتمالات نسبة الحالات الموجبة لمجموعات هذه الدراسة هي 4.50 و 4.95 على التوالي أعلى، مقارنة مع نظرائهم الأصغر سنا (15-24 سنة).

الاستنتاجات: بينما زاد اعتماد الأختبار وتقديم المشورة لحالات ألأيدز (HTC) خمسة أضعاف في المجتمع وثلاثة أضعاف تقريبا في مكان العمل، كانت معدلات إيجابية فيروس نقص المناعة البشرية أعلى بكثير في عام 2015 مقارنة مع عام 2012. يمكن استخدام بياناتنا جنبا إلى جنب مع بيانات المراقبة الأخرى لتتبع انتقال فيروس نقص المناعة البشرية في هذا السياق المحدد. واسترشادا بتقييم الأثر الصحي، تم تكييف برنامج الوقاية من فيروس نقص المناعة البشرية والسيطرة عليه بسهولة مع الوضع الحالي من خلال تحديد المحددات الاجتماعية والاقتصادية والبيئية للصحة.

الكلمات المفتاحية: تقييم الأثر الصحي، التعدين، إدارة الصحة المجتمعية، الصحة المهنية، وفيروس نقص المناعة البشرية، والأمراض المنتقلة جنسيا، وزامبيا

赞比亚西北铜矿工程以卫生影响评估为导向对 HIV/AIDS 进行预防和控制所取得的经验和教训

Astrid M. Knoblauch, Mark J. Divall, Milka Owuor, Kennedy Nduna, Harrison Ng'uni, Gertrude Musunka, Anna Pascall, Jürg Utzinger, Mirko S. Winkler

摘要

引言：为了避免或降低潜在的与工程有关的不良健康影响，赞比亚西北部 Kalumbila 的 Trident 铜矿工程委托我们开展了健康影响评估。根据当地 HIV 传播的易感性和其他采矿工程的既往经验，艾滋病是当地首要的健康问题。因此，我们制定了 HIV/AIDS 管理计划，包括社区和工作场所的干预措施。其中，艾滋病毒检测和咨询（HTC）是关键的组成部分。在此，我们报告了四年内 HTC 的数据趋势。

方法：在受 Trident 铜矿工程影响的 13 个社区中，从 2012 年起实施 HTC，采用快速诊断试剂检测，同时，由经过培训的人员进行检测前和检测后咨询。此外，与矿山开发同步，从 2013 年开始对工程中的工人开展 HTC。在研究人群中评估 HTC 的效果和 HIV 阳性率，并使用回归分析其与人口因素的相关性。

结果：随着时间的推移，总共有 11 638 名社区成员和 5 564 名工人参与了 HTC。社区的 HIV 阳性率在 2012 年为 3.0%，2015 年为 3.4%；而 2013 年工人的 HIV 阳性率为 5.2%，2015 年为 4.3%。妇女的检测结果阳性率更高（社区女性，*OR*= 1.96, 95%*CI*：1.55-2.50；女工，*OR* = 2.90, 95%*CI*：1.74-4.84）。35-49 岁年龄段的 HTC 参与者受艾滋病影响最大，社区样本平均阳性率为 6.6%，工人样本平均阳性率为 7.9%。这些研究组与 15-24 岁年龄组相比，阳性率分别有 4.50 和 4.95 的上升。

结论：虽然在社区和工地HTC参与分别增加了五倍和近三倍，但2015年HIV阳性率并不显著高于2012年。我们的数据可以与其他监测数据一起使用，以追踪艾滋病毒在这一特定环境下的传播。在健康影响评估的指导下，通过确定影响健康的社会经济和环境因素，艾滋病预防和控制项目能够较好地适应当前的环境。

Translated from English version into Chinese by Xin-Yu Feng, edited by Pin Yang

CHINESE CENTER FOR DISEASE CONTROL AND PREVENTION
NATIONAL INSTITUTE OF PARASITIC DISEASES

Expérience et leçons de l'évaluation de l'impact sur la santé guidant la prévention et le contrôle du VIH / sida dans un projet de mines de cuivre, dans le nord-ouest de la Zambie

Abstract
Contexte: Pour éviter ou atténuer les potentiels effets négatifs sur la santé liés au projet de cuivre « Trident », situé à Kalumbila dans le nord-ouest de la Zambie, une étude d'évaluation d'impact sur la santé a été commanditée par le projet. Le VIH a été identifié comme un problème de santé prioritaire en se basant sur la vulnérabilité locale à la transmission du VIH et sur l'expérience antérieure d'autres projets miniers en Afrique. Par conséquent, un plan de gestion du VIH / sida a été élaboré, incluant des interventions au niveau communautaire et du lieu de travail, avec comme éléments clés le dépistage et conseil sur le VIH (DCV). Nous présentons les tendances des données DCV sur une période de 4 ans.
Méthode: Dans 13 communautés affectées par le projet Trident, DCV a été implémenté à partir de 2012, en utilisant des tests de diagnostic rapide, accompagnés de conseils pré- et post-tests par le biais d'un personnel qualifié. En plus, DCV a été initiée dans le lieu de travail du projet en 2013, coïncidant avec le lancement du développement minier. Les taux d'absorption et de positivité du DCV ont été évalués dans la population et corrélés à des facteurs démographiques en utilisant une analyse de logistique régression.
Résultats: Au total, 11,638 membres de la communauté et 5,564 travailleurs ont fait le DCV avec une augmentation au fil du temps. Le taux de positivité du VIH dans la communauté était de 3,0% en 2012 et de 3,4% en 2015, tandis que le taux de positivité chez les travailleurs était de 5,2% en 2013 et de 4,3% en 2015. Les femmes ont montré une probabilité nettement plus élevée d'avoir un résultat positif (odds ratio (*OR*) = 1,96, 95% intervalle de confiance (*IC*) = 1,55-2,50 chez les femmes dans la communauté et *OR* = 2,90, 95% *IC* = 1,74-4,84 chez les femmes travailleurs). Les personnes dépistées dans le groupe d'âge 35-49 ans ont été les plus touchés par le VIH, avec un taux de positivité moyen de 6,6% dans l'échantillon communautaire et 7,9% dans l'échantillon des travailleurs. Ce groupe d'âge avait 4,50 et 4,95 la plus grande chance d'être positive, respectivement, comparé à leurs homologues plus jeunes âgés de 15-24 ans.
Conclusion: Alors que l'absorption de DCV a augmenté de cinq fois dans la communauté et presque trois fois chez les travailleurs, les taux de positivité du VIH étaient insignifiants plus élevés en 2015 qu'en 2012. Nos données peuvent être utilisées aux côtés d'autres données de surveillance pour suivre la transmission du VIH dans ce contexte spécifique. Guidé par l'évaluation d'impact sur la santé, le programme de prévention et de lutte contre le VIH a été facilement adapté au contexte actuel en identifiant les déterminants socio-économiques et environnementaux de la santé.
Mots-clés: Évaluation d'impact sur la santé, exploitation minière, gestion de la santé communautaire, santé au travail, VIH, infections sexuellement transmissibles, Zambie.

Опыт и уроки оценки воздействия на здоровье, направленные на предотвращение и борьбу с ВИЧ / СПИДом в проекте по разработке месторождений меди, северо-западная часть Замбии

Абстракт
Введение: Чтобы избежать или смягчить потенциальные неблагоприятные последствия для здоровья,проект по добыче меди 'Тридент' в Калумбила, в северо-западной части Замбии, выдал мандат на оценку воздействий проекта на здоровье. Основываясь на местной уязвимости к переносу ВИЧ и опыте прошлых горнодобывающих проектов в Африке, ВИЧ был идентифицирован как приоритетная проблема здоровья. Таким образом, был разработан план управления ВИЧ/СПИД в общинах и среди рабочих, включающий привентивные меры, в которых тестирование на ВИЧ и консультирование (ВТК) является одним из ключевых компонентов. Мы представляем тенденции в данных ВТК за 4-летний период.
Методы: В 13 общинах, затронутых проектом 'Тридент', ВТК было реализовано с 2012 года с использованием быстрых диагностических тестов в сопровождении с до- и после- тестовым консультированием квалифицированным персоналом. Кроме того, ВТК было начато среди рабочих в 2013 году, что совпало с началом развития шахты. Обращение в ВТК и частота положительных результатов были оценены в исследуемой популяции и связаны с демографическими факторами используя регрессионный анализ.
Результаты: В общей сложности, 11,638 членов общины и 5,564 рабочих обратились в ВТК, с ростом количества людей в течении времени. ВИЧ-позитивность в сообществе составила 3,0% в 2012 и 3,4% в 2015 годах, в то время как ВИЧ-положительность среди рабочих составила 5,2% в 2013 году и 4,3% в 2015 году. Женщины показали значительно более высокие шансы иметь положительный результат теста (отношение шансов (Ош) = 1,96, 95% доверительный интервал (ДИ) 1,55-2,50 среди женщин в общине и Ош = 2,90, 95% ДИ 1,74-4.84 среди женщин в составе рабочей силы). Пользователи ВТК в возрастной группе 35-49 лет были наиболее затронуты ВИЧ: средняя частота положительных составляет 6,6% в выборке из сообщества и 7,9% в выборке из рабочей силы. Эти группы имели 4.50 и 4.95 более высокие шансы быть позитивным, соответственно, по сравнению с более молодыми представителями (15-24 лет).

Выводы: Несмотря на то, что обращение в ВТК увеличилось в пять раз в сообществе и почти трехкратно на рабочем месте, позитивность ВИЧ была незначительно выше в 2015 году, по сравнению с 2012. Наши данные могут использоваться вместе с другими данными наблюдения для отслеживания передачи ВИЧ в этом конкретном контексте. Руководствуясь оценкой воздействия на здоровье, программа профилактики и контроля ВИЧ была адаптирована к текущей ситуации путем выявления социально-экономических и экологических факторов здоровья.
Ключевые слова: Оценка воздействия на здоровье; добыча полезных ископаемых; управление здравоохранением в сообществе; охрана здоровья на рабочем месте; ВИЧ; инфекции передающиеся половым путем; Замбия.

Experiencia y lecciones de la evaluación del impacto sanitario que orientan la prevención y el control del VIH / SIDA en un proyecto de mina de cobre en el noroeste de Zambia

Abstracto

Introducción: Para evitar o mitigar posibles efectos negativos en salud producidos por el desarrollo de los proyectos, el proyecto de extracción de cobre "Trident" en Kalumbila, ha llevado a cabo una evaluación de impacto del mismo. Debido a la vulnerabilidad local ante la transmisión del VIH y experiencias previas en otros proyectos de actividad minera en África, el abordaje del VIH se ha identificado como una cuestión prioritaria en salud. Por esta razón se ha desarrollado un plan para el manejo de VIH/SIDA, incluyendo intervenciones a nivel comunitario y en centros de salud, siendo el test y la consejería relativa a VIH (HTC por sus siglas en inglés) componentes clave de estas intervenciones. En este artículo presentamos los datos sobre las tendencias en HTC durante un periodo de 4 años.

Métodos: La estrategia HTC ha sido implementada en 13 comunidades incluidas en el proyecto Trident desde el año 2012, usando test rápidos de diagnóstico acompañados de un asesoramiento pre y post-test realizado por personal entrenado. Además, en el año 2013 se inició la estrategia HTC en los proyectos para la población trabajadora, coincidiendo con el lanzamiento del desarrollo minero. Se evaluó el uso de HTC, así como la prevalencia de VIH a través de un estudio poblacional relativo a factores demográficos, usando un análisis regresivo.

Resultados: Un total de 11.638 miembros de las comunidades y 5.564 trabajadores han utilizado los servicios de HTC, con una tendencia creciente a lo largo del tiempo. La prevalencia de VIH en las comunidades fue de 3% en 2012 y 3,4% en 2015, mientras que la prevalencia en la población trabajadora fue de 5,2% en 2013 y 4,3% en 2015. En relación a la probabilidad de obtener un resultado del test positivo, las mujeres presentaron un odds significativamente mayor (odds ratio (*OR*)=1,96, 95% intervalo de confianza (*IC*) 1,55-2,50 entre las mujeres de las comunidades y *OR*=2,90, 95% *IC* 1,74-4,84 entre las mujeres de la población trabajadora). Entre los usuarios de HTC, el grupo de edad comprendido entre 35-49 años fue el más afectado por el VIH, con una prevalencia media de 6,6% en la muestra de la población de las comunidades y 7,9% en la muestra de la población trabajadora. Este grupo de estudio tuvo una razón de productos (odds ratio) de obtener un resultado positivo del test de 4,50 y 4,95 respectivamente, en relación a sus equivalentes más jóvenes (15-24 años).

Conclusiones: Mientras la adopción de HTC aumentó cinco veces en las comunidades y casi tres veces en la población trabajadora, la prevalencia de VIH fue significativamente mayor en 2015 en comparación con 2012. Junto con datos de vigilancia epidemiológica, nuestros datos pueden ser útiles para monitorizar la transmisión de VIH en este contexto específico. Guiado por la evaluación de impacto en salud, el programa de prevención y control del VIH fue fácilmente adaptado al escenario actual a través de la identificación de determinantes de salud socioeconómicos y medioambientales.
Palabras clave: Evaluación de impacto en salud, actividad minera, manejo de enfermedades en la comunidad, salud laboral, VIH, infecciones de transmisión sexual, Zambia.
