# Supplementary material for: Experience and lessons from health impact assessment guiding prevention and control of HIV/AIDS in a copper mine project, northwestern Zambia
Source: Infect Dis Poverty. 2017 Jul 4;6:114. doi: 10.1186/s40249-017-0320-4 (PMC5496403; doi:10.1186/s40249-017-0320-4)
Supplement: Supplementary file 2 — HIV positivity rate of first-time testers in community members and workforce aged 15–49 years, Trident project area, 2012–2015. (DOCX 44 kb) [file 40249_2017_320_MOESM2_ESM.docx]

**Additional file 1: HIV positivity rate of first-time testers in community members and workforce aged 15-49 years, Trident project area, 2012-2015**

| **Year** | **2012** | **2013** | **2014** | **2015** |
| --- | --- | --- | --- | --- |
| **Community (*n*)** | **326** | **914** | **434** | **1 478** |
| Average overall HIV positivity rate (%; 95% *CI*) | 3.4 (1.7-6.0) | 3.1 (2.0-4.4) | 4.1 (2.5-6.5) | 3.2 (2.3-4.2) |
| **Workforce (*n*)** | - | **395** | **705** | **290** |
| Average overall HIV positivity rate (%; 95% *CI*) | - | 6.1 (3.9-8.9) | 4.1 (2.8-5.9) | 5.9 (3.5-9.2) |
| *CI*, confidence interval; -, no data available | | | |  |
